# Less Positive Parenting Appears to be a Consequence, Rather Than a Cause, of Youth Antisocial Behavior: Results from a Longitudinal Twin Study

**DOI:** 10.1007/s10802-026-01425-2

**Published:** 2026-02-14

**Authors:** Alaina M. Di Dio, Elizabeth A. Shewark, Luke W. Hyde, S. Alexandra Burt

**Affiliations:** 1 Department of Psychology and Neuroscience, University of Colorado Boulder, Boulder, CO USA; 2 Institute for Behavioral Genetics, University of Colorado Boulder, Boulder, CO USA; 3 Department of Psychology, University of Notre Dame, Notre Dame, IN USA; 4 Department of Psychology, University of Michigan, Ann Arbor, MI USA; 5 Department of Psychology, Michigan State University, East Lansing, MI USA

**Keywords:** Parent–child relationship, Positive parenting, Antisocial behavior, Twin differences

## Abstract

Although positive parenting in childhood consistently predicts less adolescent antisocial behavior (ASB) over time, the etiology and direction of their association remain unclear. To fill this gap, we sought to illuminate prospective associations and potential changes in the etiology of the relationship between maternal positive parenting and youth ASB from middle childhood to adolescence using a cross-lagged, twin differences design. Participants were drawn from a longitudinal study with planned missingness (1,422 twins ages 6–11 at Wave 1 and 852 twins ages 11–19 at Wave 2) within the Michigan State University Twin Registry. Phenotypic analyses indicated that more childhood ASB significantly predicted less positive parenting in adolescence, but not the reverse. Twin difference-score analyses similarly revealed that, within both monozygotic (MZ) and dizygotic (DZ) twin pairs, co-twin differences in the positive parenting received in middle childhood did not significantly predict differences in adolescent ASB. However, DZ (but not MZ) co-twin differences in childhood ASB significantly predicted differences in positive parenting during adolescence, whereby the DZ co-twin exhibiting more ASB in childhood received less positive parenting in adolescence. These results collectively suggest a child-driven, genetically mediated effect of early ASB on later positive parenting, such that genetic influences on ASB in middle childhood appear to evoke reductions in positive parenting during adolescence. Moreover, because effect sizes did not differ across zygosity, results also suggest that shared familial/environmental confounds at least partially underlie the association. These findings have important implications for interventions and etiologic models of youth ASB across development.

**Supplementary Information:**

The online version contains supplementary material available at 10.1007/s10802-026-01425-2.

The parent-child relationship is central to children’s emotional, cognitive, and behavioral development (Bowlby, 1969, 1973; Baumrind, 1966; Maccoby & Martin, 1983). Whereas negative and harsher forms of parenting are robustly linked to adverse child outcomes (e.g., Amato & Fowler, 2002), positive parenting (typically encompassing warmth/nurturance, positive behavioral management, sensitivity/responsiveness, and involvement/support; e.g., Prime et al., 2023; Sanders et al., 2014), supports children’s development and well-being. For example, maternal nurturance is linked to the development of key socioemotional competencies and is positively associated with children’s academic achievement, psychological adjustment, and self-worth/self-esteem (Goering & Mrug, 2023; Pinquart, 2015; Buri et al., 1992; Khaleque, 2013).

Positive parenting is also associated with child psychopathological outcomes, including antisocial behavior (ASB). ASB is defined as actions that transgress societal norms and infringe upon others’ rights, encompassing both aggressive and rule-breaking behaviors (Burt, 2012). In phenotypic studies, higher levels of positive parenting behaviors, such as warmth, nurturance, behavioral control, involvement, sensitivity, and support, are consistently associated with less youth ASB cross-sectionally and longitudinally (Rothenberg et al., 2020; Arim et al., 2011; Pardini et al., 2007; Pinquart, 2017; Álvarez-García et al., 2016; Van Heel et al., 2019; Cooke et al., 2022), suggesting that positive parenting may protect against the development of youth ASB.

Researchers have frequently interpreted these findings within a “parent effects model,” in which parenting causally *exacerbates* or *inhibits* child ASB (Collins et al., 2000; Maccoby, 2000). Consistent with this interpretation, many family-based interventions for youth ASB aim to decrease harsh/negative parenting and strengthen positive parenting (Fagan & Benedini, 2016). Nonetheless, it is now recognized that the parent–child relationship can also be (at least in part) a consequence of the child’s behavior. One framework for understanding bidirectional associations in the context of child ASB is coercion theory (Patterson, 1982; Patterson et al., 1989), which describes a cycle of escalating parent–child interactions in which aversive child behaviors (e.g., noncompliance, tantrums) and harsh/inconsistent parenting (e.g., yelling, giving in after misbehavior) are *mutually* reinforced, ultimately contributing to the development and maintenance of child ASB. In this way, parents not only shape but can also *respond* to their child’s ASB by acting harsher and less nurturing (e.g., Anderson et al., 1986; Serbin et al., 2015). One mechanism through which such child-driven effects may emerge are evocative gene–environment correlations (*r*GE). Evocative *r*GE are genetically influenced exposures to environmental experiences, whereby children’s heritable characteristics evoke reactions from others in their environment consistent with their genetic predispositions (Scarr & McCartney, 1983). In the case of positive parenting and ASB, evocative *r*GE would manifest as less parental warmth toward their children in response to their children’s *genetically influenced* ASB.

A small handful of cross-sectional studies have employed genetically informed designs to examine child effects/evocative *r*GE and parent effects in the etiology of associations between positive parenting and ASB. Some have suggested that this relationship is genetically mediated and reflective of evocative *r*GE. For example, in a small adoption study, Ge et al. (1996) examined adoptees (ages 12 to 18) and their adoptive parents, finding that children at a higher genetic risk for externalizing psychopathology (based on externalizing psychopathology in their biological parents) received less nurturance/involvement and harsher discipline than adoptees who were not at genetic risk. Other studies pointed to the presence of at least some environmental mediation as well (Di Dio et al., 2025; Buschgens et al., 2010; Zhou et al., 2025). For instance, Di Dio et al. (2025) examined the etiology of associations between positive parenting (assessed via the same measure examined herein) and ASB in middle childhood using a discordant twin design, finding evocative *r*GE on maternal positive parenting and shared environmental influences (i.e., factors that increase similarity between co-twins regardless of their genetic relatedness) on the association between ASB and parental positive parenting more broadly.

Unfortunately, most behavioral genetic studies in this area have been cross-sectional and focused on childhood, and thus, we know relatively little about the direction of effects over time, particularly in adolescence. Indeed, although longitudinal genetically informed studies from early to middle childhood have generally found parent-driven environmental effects of early positive parenting behaviors (e.g., warmth, Reuben et al., 2016; maternal expressed emotion, Caspi et al., 2004; warmth and sensitivity, Boeldt et al., 2011) on child ASB, in an adoption study, Marceau et al. (2025) found that declines in paternal (but not maternal) warmth throughout childhood were associated with increases in child ASB from early- to mid-adolescence, results that may reflect both child-driven evocative effects and parent-driven environmental influences.

The relative absence of longitudinal genetically informed studies later in development represents a critical gap in the literature, as both parenting and ASB evolve in important ways. Relative to childhood, adolescence is characterized by increases in ASB (e.g., see Carroll et al., 2023 for a review) and decreases in positive parenting behaviors, such as warmth, closeness, and support (e.g., Marceau et al., 2015; Zheng & McMahon, 2022). Consequently, the direction of effects between youth ASB and maternal positive parenting, its etiologic origins, and whether/how the associations observed in childhood persist into adolescence remain unclear.

The goal of the current study was to elucidate both the direction and etiology of the association between maternal positive parenting and youth ASB from middle childhood to adolescence using a twin differences design. By leveraging naturally occurring differences in environmental experiences and segregating genes shared (100% or an average of 50%), the twin differences design offers a powerful approach for disentangling genetic and environmental contributions to longitudinal associations. Monozygotic (MZ) twins are genetically identical and differ only due to child-specific/nonshared environmental influences (e.g., differential parenting) and measurement error. Differences between MZ twins thus cannot be confounded by shared genes or shared family environments, allowing researchers to explicitly evaluate whether sibling differences in parenting are *environmentally linked* to changes in youth ASB. Dizygotic (DZ) twins, by contrast, differ due to these child-specific environmental experiences but also the (on average) 50% of their genes the twins do not share (Plomin et al., 2008). Thus, examining discordant DZ twins enables researchers to evaluate whether twin differences in parental treatment are *genetically linked* to later youth ASB. Using this twin differences design, and based on prior cross-sectional work in middle childhood and adolescence (Di Dio et al., 2025; Ge et al., 1996), we hypothesized that the longitudinal association between maternal positive parenting and youth ASB would reflect a child-driven, genetically mediated association, such that children’s genetically influenced ASB evokes less positive parenting from childhood into adolescence.

## Methods

### Participants

Families were drawn from the Twin Study of Behavioral and Emotional Development in Children (TBED-C), a study within the Michigan State University Twin Registry (MSUTR; Burt & Klump, 2019). This study includes a population-based arm (*N* = 528 families; MZ *n* = 260, DZ *n* = 268) and an under-resourced arm living in neighborhoods with higher-than-average poverty levels (*N* = 502 families; MZ *n* = 166, DZ *n* = 336). Participants were recruited through birth records (see Burt & Klump, 2019 for descriptions of the participation rate, design, and recruitment). All procedures were approved by the Institutional Review Board of Michigan State University. Children provided informed assent, and parents provided informed consent for themselves and their children. Participants were 49% female (sex assigned at birth), and their racial identities were 82% White, 10% Black, 1% Asian, 1% indigenous, and 6% multiracial.

The current study utilizes Wave 1 data from participants residing in moderately-to-severely disadvantaged neighborhoods (*N* = 757 families, MZ *n* = 287; DZ *n* = 470), a portion of whom were reassessed as part of a longitudinal follow-up in the Michigan Twin Neurogenetics Study (MTwiNS). Because our follow-up assessment included neuroimaging, we were only able to fund the reassessment of 475 families. However, the response rate among eligible families was 90.20%, of which 80.40% agreed to participate. A small number of pairs (*n* = 45 twin pairs) were aged 7 to 10 or 20 to 25 at the Wave 2 assessment and were excluded from analyses to restrict the sample to adolescence. Final sample sizes were 711 twin pairs at Wave 1 (MZ *n* = 268; DZ *n* = 443; 6 to 11 years old, *M* age = 7.93, *SD* = 1.48) and 426 twin pairs at Wave 2 (MZ *n* = 162; DZ *n* = 264; 11 to 19 years old, *M* age = 15.35, *SD* = 1.94).

### Measures

#### Children’s ASB

At both assessments, mothers completed the Achenbach Child Behavior Checklist (CBCL; Achenbach & Rescorla, 2001) for each twin. We used the Externalizing Behavior Scale (35 items; Waves 1 and 2 *α*s = 0.90 and 0.88, respectively) to assess children’s aggressive (e.g., destroys people’s things, fights, threatens people, argues) and rule-breaking (e.g., lies, breaks rules, steals, truant) behaviors. Mothers rated the extent to which statements described the child’s behavior over the last six months on a three-point scale (0 = “*never”* to 2 = “*often/mostly true”*).

## Maternal Positive Parenting

Maternal positive parenting was measured via the Parent-Child Involvement scale on the Parental Environment Questionnaire (PEQ), a measure that has demonstrated good internal consistency (*α* = 0.74–0.79) and evidence of validity (e.g., *r* = .50–0.59 with the Cohesion subscale of the Family Environment Scale; Moos & Moos, 1986) in both middle childhood and adolescence (Elkins et al., 1997). Whereas the only longitudinal behavioral genetic study from middle childhood to adolescence assessed only parental warmth (Marceau et al., 2025), the PEQ Involvement scale evaluated multiple facets of positive parenting, including affective behaviors (e.g., support, closeness, nurturance) and positive behavioral reinforcement (i.e., praise). To avoid shared informant effects, in which the same informant reports on the predictor and outcome variables, we examined twin self-reports of the maternal positive parenting received. Each twin individually rated the parenting received from their mother (12 items; See Table S1 for the wording of all scale items; Waves 1 and 2 *α*s = 0.71 and 0.88, respectively). Each item was rated on a four-point scale from 1 = *definitely true* to 4 = *definitely false* and coded such that higher scores indicated higher levels of positive parenting. At Wave 1, the PEQ was read to twins with reading levels under fifth grade (as assessed with a brief reading screen; Torgesen et al., 1999) to assume comprehension.

Confirmatory factor analyses (CFA) were conducted to evaluate the factor structure of the PEQ parenting measure. CFA results suggested that a single factor with estimated loadings for each of the 12 items fit the data well, but after excluding the three items with the lowest loadings, the 9-item model demonstrated excellent fit in both middle childhood and adolescence. See Table S1 for the fit of all CFAs and their factor loadings. Additionally, and importantly, most twin pairs differed in the parenting received, with only 7–11% of co-twins reporting identical levels of parenting at either assessment. Information regarding the magnitude of co-twin differences compared to unrelated individuals in the sample can be found in Supplemental Materials.

## Missing Data

Given the attrition from Wave 1 to Wave 2 (~ 40%), we evaluated whether the parenting and ASB data were missing completely at random (MCAR) and whether twins who participated only in Wave 1 differed from twins who participated in both Waves. Little’s (1988) MCAR test was nonsignificant (χ^2^ (18) = 27.20, *p* = .074), and there were no significant group differences in parenting, child ASB, or socioeconomic indices (*p*s > 0.05). Additional information regarding these analyses is available in Supplemental Materials, with results presented in Tables S2–S3.

## Statistical Analyses

Phenotypic and twin difference-score analyses were conducted to examine the direction and etiology of the association between maternal positive parenting and youth ASB. We first confirmed phenotypic/individual-level associations by fitting a cross-lagged model to the phenotypic data. A conceptual model is presented in Figure S1. Cross-age but within-trait partial regression coefficients index the stability of parenting and ASB from middle childhood to adolescence (i.e., *b*3, *b*4). Cross-lagged partial regression coefficients indicate whether positive parenting and children’s ASB independently influenced each other from childhood to adolescence (allowing us to examine the direction of effects), controlling for the stability and cross-lagged contributions of the other trait (i.e., *b*1, *b*2). Finally, correlation coefficients computed at both timepoints revealed age-specific correlations between positive parenting and youth ASB (i.e., *r*1, *r*2). These analyses were conducted across the full sample using hierarchical clustering with robust standard errors to account for the data’s nested structure (i.e., twins and mothers were nested within families). As is recommended with cluster-robust standard errors, we utilized Maximum Likelihood Robust estimation to handle missing data (McNeish et al., 2017).

We next fit this model using co-twin difference scores in place of the phenotypic data. We first computed co-twin difference scores (i.e., Twin A’s score – Twin B’s score, where twins were randomly assigned to Twin A or B), separately by zygosity, for parenting and ASB at both timepoints. Because MZ and DZ co-twins both share 100% of their rearing environment but differ in their proportion of segregating genes (DZ ~ 50%; MZ = 100%), we can compare the pattern of twin difference findings across zygosity to infer the etiology of the longitudinal association. MZ difference scores control for both shared environmental and genetic effects, providing a direct estimate of nonshared environmental influences. Thus, any (statistically significant) cross-lagged association greater than zero in MZ twins would be indicative of nonshared environmental mediation (see Scenario 1 in Figure S2; McGue et al., 2010). By contrast, co-twin difference scores larger than zero within DZ pairs are a function of nonshared environmental influences and the ~ 50% of genes they do not share. Consequently, if we observe significant prospective associations in DZ but not MZ co-twins, and the magnitude of the association is significantly greater in DZ twins, we would conclude that the longitudinal association is genetically mediated (Scenario 2). Importantly, because this is a child-based twin study, the presence of genetic influences would necessarily reflect an *r*GE process, as genetic influences represent the impact of the child’s genes on parenting (see Klahr & Burt, 2014). By contrast, if we observe cross-lagged associations that are equivalent in magnitude and significantly different from zero in *both* MZ and DZ pairs, we would conclude that the relationship is nonshared environmental in origin (Scenario 1). Finally, if difference-score associations are observed only at the phenotypic level but are not significantly different from zero in either MZ or DZ pairs, and/or if the magnitude of the association in DZ twins is not significantly greater than that in MZ twins, we would conclude that the association is due to both genetic and shared environmental effects (Scenario 3).

With this interpretative framework in mind, we fit a series of cross-lagged models at the family level using the difference scores. Cross-age but within-trait partial regression coefficients index the stability of co-twin differences in parenting and ASB. Cross-lagged partial regression coefficients index the extent to which twin differences in positive parenting and children’s ASB independently influenced one another from childhood to adolescence, controlling for the stability and cross-lagged contributions of twin differences in the other trait. Finally, correlations computed at each timepoint index age-specific associations between twin differences in parenting and youth ASB. Because our models included autoregressive and cross-lagged paths, correlations functioned as residuals in adolescence, allowing us to discern adolescence-specific associations between twin differences in parenting/ASB independent of childhood associations.

This model was first fit separately for MZ and DZ co-twin difference scores. We then fit a third model that constrained all parameter estimates to be equal across MZ and DZ pairs for a stronger test of the association’s etiology, although means and variances of the scores were free to vary. As a final step, we repeated our twin difference analyses amongst those twin pairs most discordant for ASB (top 25% of sample; co-twins differing by at least one standard deviation; *N* = 196 twin pairs (MZ *n* = 49, DZ *n* = 147) at Wave 1 and *N* = 116 twin pairs (MZ *n* = 29, DZ *n* = 87) at Wave 2. In this way, we could ascertain whether results from the full sample persisted in those twin pairs exhibiting the most pronounced differences in ASB.

Finally, because our Wave 1 and Wave 2 data were collected on average 7.51 years apart, we conducted sensitivity analyses to examine whether results varied by the time interval between assessments. We divided co-twins with Wave 2 data into two groups based on the median observed lag time (i.e., 7.27): co-twins reassessed at Wave 2 *within* 7.27 years of their initial assessment (*n* = 418) and those reassessed *more than* 7.27 years after their Wave 1 assessment (*n* = 434). The median lag time was used as the cutoff because it produced groups of approximately equal sizes. We then fit a constraint model that equated parameter estimates across groups to test whether phenotypic associations were consistent across shorter and longer intervals.

All models were fit in Mplus (version 8.7; Muthén & Muthén, 2021). Prior to analyses, ASB data were log-transformed to address positive skewness, and participants’ sex was regressed out of the raw parenting and log-transformed ASB scores. All cross-lagged models were conducted using the full 12-item and reduced 9-item positive parenting scales. Results were nearly identical across both operationalizations, so models using the 9-item version are reported in the main text (given its excellent fit to the data), while those using the full 12-item version are presented in Table S4 (for the correlations), Figures S3–S7 (for the cross-lagged models), and Figure S8 (for the sensitivity analyses). Model fit was assessed using four indices: Chi-Square (χ^2^), Root Mean Square Error of Approximation (RMSEA), Comparative Fit Index (CFI), and Standardized Root Mean Squared Residual (SRMR). A nonsignificant χ^2^ value and RMSEA and SRMR values of less than 0.06 and 0.05, respectively, indicate adequate model fit, while CFI values of 0.95 or above indicate excellent model fit (Hooper et al., 2008). For the twin difference-score models, Full-Information Maximum-Likelihood estimation was used to estimate missing data, which can handle large amounts of missing data (Enders, 2001, 2023).

## Results

### Descriptive Statistics and Correlations

Descriptive statistics for ASB and positive parenting across the full sample, and twin differences in those scores, are presented in Table S5. Both phenotypic and twin difference-score correlations are presented in Table S6. Phenotypic correlations revealed that maternal positive parenting was cross-sectionally and negatively associated with youth ASB at both timepoints, and that both traits demonstrated significant stability from childhood to adolescence. Although childhood ASB was negatively and significantly associated with adolescent positive parenting, childhood positive parenting was not significantly associated with adolescents’ ASB. This overall pattern persisted to the twin difference scores, in that childhood co-twin differences in parenting were not significantly associated with differences in ASB during adolescence. Additionally, DZ (but not MZ) co-twin differences in childhood ASB were negatively and significantly associated with co-twin differences in parenting during adolescence, and the magnitude of this association was notably smaller in MZ compared to DZ twin pairs (*p* = .070).

## Cross-Lagged Models

### Phenotypic

**Fig. 1:**
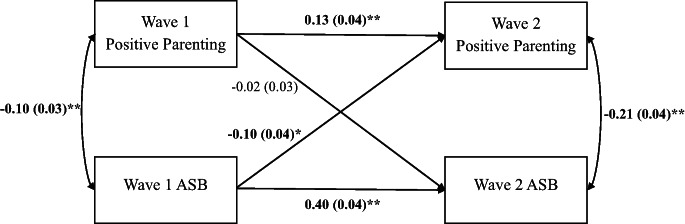
Phenotypic Cross-Lagged Model of Maternal Positive Parenting and Youth ASB. *N *= 1,422. Fit indices: χ^2^(0) = 0.00, *p *< .001, root mean square error of approximation (RMSEA) = 0.00, comparative fit index (CFI) = 1.00, standardized root mean square residual (SRMR) = 0.00. The observed variables are maternal positive parenting (9-item version) and log-transformed youth ASB scores across the full sample at Wave 1 (middle childhood) and Wave 2 (adolescence). Unstandardized regression coefficients are presented for the single-headed arrows (i.e., paths), and correlations are presented for the double-headed arrows. Standard errors for the estimates are presented in parentheses. Significant estimates are bolded. ** *p *< .01 and * *p *< .05, two-tailed

Figure 1 presents the path diagram for the phenotypic associations. Fit indices indicated that this model was, as expected, just identified. Both positive parenting and ASB demonstrated significant within-trait stability across time. In middle childhood and adolescence, higher levels of positive parenting were significantly correlated with less youth ASB. Additionally, higher levels of ASB in childhood significantly predicted reduced positive parenting in adolescence. Critically, however, parenting in childhood did not significantly predict adolescent ASB.

### Twin Differences

**Fig. 2:**
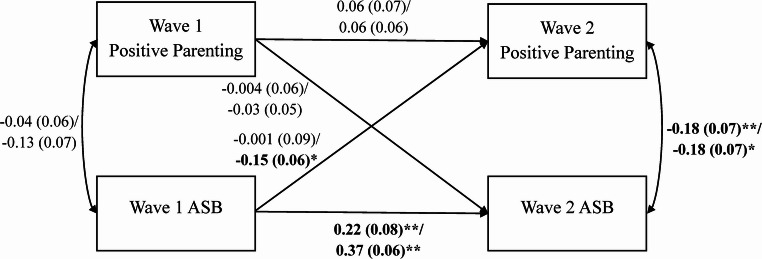
Cross-Lagged Model of Twin Differences in Maternal Positive Parenting and Twin Differences in Youth ASB in MZ and DZ Twin Pairs. Monozygotic (MZ) *N *= 268 pairs; Dizygotic (DZ) *N *= 443 pairs. Fit indices for both the MZ and DZ twin difference-score models: χ^2^(0) = 0.00, *p *= .000, root mean square error of approximation (RMSEA) = 0.00, comparative fit index (CFI) = 1.00, standardized root mean square residual (SRMR) = 0.00. The observed variables are MZ and DZ twin difference scores in maternal positive parenting (9-item version) and log-transformed youth ASB at Wave 1 (middle childhood) and Wave 2 (adolescence). Path and correlation estimates are presented first for the MZ model and then just below for the DZ model (MZ estimate (*SE*) / DZ estimate (*SE*)). Unstandardized regression coefficients are presented for the single-headed arrows (i.e., paths), and correlations are presented for the double-headed arrows. Standard errors for the estimates are presented in parentheses. Significant estimates are bolded. ** *p *< .01 and * *p *< .05, two-tailed

The path diagrams for the full, unselected samples of MZ and DZ twins are presented in Fig. 2. Results were nearly identical across MZ and DZ pairs, except for one cross-lagged path. Twin differences in ASB, but not parenting, evidenced significant stability over time in both MZ and DZ twin pairs. We also observed cross-sectional associations between co-twin differences in parenting and youth ASB in MZ and DZ twins, but these associations were only significant during adolescence. Once again, neither MZ nor DZ co-twin differences in parenting during childhood significantly predicted twin differences in adolescent ASB. By contrast, twin differences in childhood ASB negatively predicted co-twin differences in adolescent positive parenting, but this association was only significant in DZ twins. However, the magnitude of the DZ association was not quite significantly greater than that in MZ co-twins (*p* = .053).

**Fig. 3:**
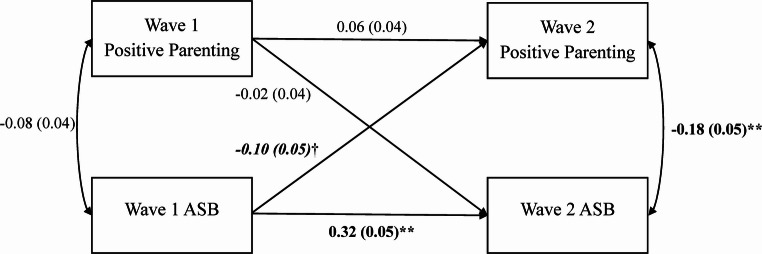
Cross-lagged Constraint Model of Twin Differences in Maternal Positive Parenting and Twin Differences in Youth ASB. *N *= 711 pairs; Monozygotic (MZ) *n *= 268 pairs, Dizygotic (DZ) *n *= 443 pairs. Fit indices: χ^2^ (6) = 4.53, *p *= .606, root mean square error of approximation (RMSEA) = 0.00, comparative fit index (CFI) = 1.00, standardized root mean square residual (SRMR) = 0.03. The observed variables are MZ and DZ twin difference scores in maternal positive parenting (9-item version) and log-transformed youth ASB at Wave 1 (middle childhood) and Wave 2 (adolescence). Unstandardized regression coefficients are presented for the single-headed arrows (i.e., paths), and correlations are presented for the double-headed arrows. Standard errors for the estimates are presented in parentheses. Significant estimates are bolded. ** *p *< .01, * *p *< .05, and †*p *< .10, two-tailed

The constraint model for the full sample evidenced excellent model fit (χ^2^ (6) = 4.53, *p* = .606; RMSEA = 0.00; CFI = 1.00; SRMR = 0.03), suggesting that the parameter estimates do not significantly differ between MZ and DZ twins and can thus be constrained to be equal (see Fig. 3). All significant paths and correlations in the constraint model generally replicated those described above. Of note, twin differences in ASB in middle childhood predicted differences in adolescent positive parenting (and not the reverse), although this association was significant only at the trend level (*p* = .051). That said, this association was negatively signed, suggesting that the co-twin engaging in more ASB in childhood received less positive parenting as an adolescent.

**Fig. 4:**
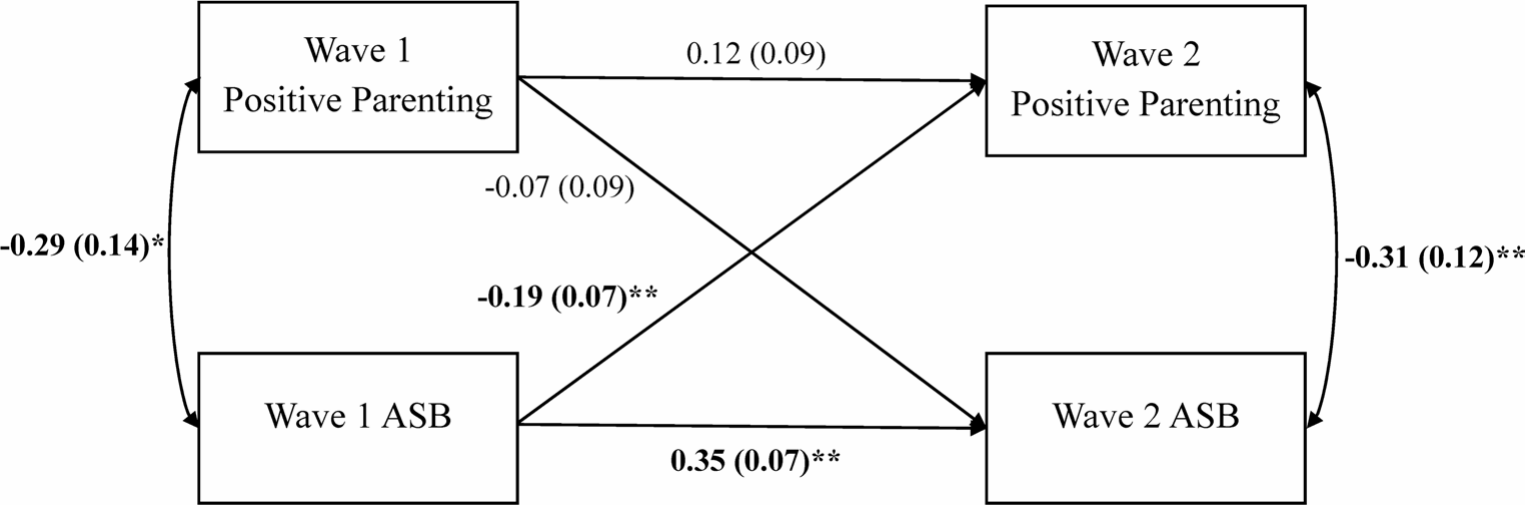
Cross-Lagged Constraint Model of Twin Differences in Maternal Positive Parenting and Twin Differences in Youth ASB for Twin Pairs Most Discordant on ASB (top 25%). *N *= 196 pairs; Monozygotic (MZ) *n *= 49 pairs, Dizygotic (DZ) *n *= 147 pairs. Fit indices: χ^2^ (6) = 3.00, *p *= .808, root mean square error of approximation (RMSEA) = 0.00, comparative fit index (CFI) = 1.00, standardized root mean square residual (SRMR) = 0.05. The observed variables are MZ and DZ twin difference scores in maternal positive parenting (9-item version) and log-transformed youth ASB at Wave 1 (middle childhood) and Wave 2 (adolescence). Unstandardized regression coefficients are presented for the single-headed arrows (i.e., paths), and correlations are presented for the double-headed arrows. Standard errors for the estimates are presented in parentheses. Significant estimates are bolded. ** *p *< .01 and * *p *< .05, two-tailed

Results in those twin pairs most discordant for ASB replicated those reported above (see Figure S9). Once again, the association between childhood ASB and adolescent positive parenting was significant in DZ (but not MZ) pairs, but its magnitude did not significantly differ between MZ and DZ twins (*p* = .182). As before, the constraint model (see Fig. 4) demonstrated excellent model fit: χ^2^ (6, *N* = 196) = 3.00 (*p* = .808), RMSEA = 0.00, CFI = 1.00, SRMR = 0.05. Results from the constraint model in the most discordant sample were similar to those in the full, unselected sample, although generally stronger in magnitude. Namely, MZ and DZ twin differences in youth ASB in middle childhood negatively and significantly predicted differences in the parenting received in adolescence, such that the twin engaging in higher levels of ASB in childhood received less positive parenting in adolescence. The path from childhood parenting to adolescent ASB, however, remained small and not significantly different than zero.

### Sensitivity Analyses

A path diagram for the phenotypic cross-lagged model constraining associations across twins assessed at Wave 2 *within* versus *after* 7.27 years of their Wave 1 assessment is presented in Figure S10. This constraint model evidenced excellent model fit (χ^2^ (6, *N* = 852) = 7.10 (*p* = .312), RMSEA = 0.02, CFI = 1.00, SRMR = 0.02), suggesting that associations were consistent across assessment intervals. Notably, and as in the primary analyses, childhood ASB negatively and significantly predicted adolescent positive parenting (but not the reverse).

## Discussion

The present study evaluated the direction and etiology of the association between maternal positive parenting and youth ASB from middle childhood to adolescence utilizing a cross-lagged, twin differences design. Our results revealed three main findings. Across all phenotypic and twin difference analyses, the cross-lagged path from childhood positive parenting to adolescent ASB was universally nonsignificant. By contrast, childhood ASB negatively and significantly predicted adolescent positive parenting in both individual level and twin difference-score models (and particularly DZ twin difference models) and did so over and above preexisting associations between the traits and their stabilities across time.

These results collectively suggest that the association between maternal positive parenting in childhood and adolescent ASB is consistent with a child-driven pathway, whereby children with higher levels of ASB in childhood elicit less positive parenting from their mothers over time. At the family level, a small but statistically significant difference-score association was observed in DZ twin pairs, indicating that the DZ co-twin who engaged in more ASB in middle childhood evoked less positive parenting from their mother years later, while the cross-lagged association in MZ twin pairs was nonsignificant and practically zero in magnitude, regardless of the severity of the sample examined. This pattern of results aligns with Scenario 2 and suggests that the association functions via genetic/evocative *r*GE mechanisms, such that mothers seemed to engage in less positive parenting *in response* to their child’s earlier genetically influenced misbehavior. That said, our results also point to shared environmental influences (Scenario 3). This is because the child-driven difference-score path coefficient in the DZ-only model was not significantly greater than that in the MZ-only model (in the unselected or most discordant sample). Furthermore, this coefficient could be constrained to be equal in MZ and DZ pairs, and both constraint models demonstrated excellent model fit. Collectively, this pattern of findings is most consistent with a child/family effects pathway, rather than a *causal* effect of parenting on youth ASB. Finally, although results were consistent across both samples, effects were stronger among the sample of co-twins most discordant on ASB (top ~ 25% of sample). These results suggest that the evocative effects of child ASB on positive parenting may be more pronounced in children exhibiting greater ASB.

Our finding of likely child-driven effects of youth ASB on positive parenting aligns with the broader developmental literature highlighting children as actively involved in shaping the parenting they receive (e.g., Yan et al., 2021), as well as studies identifying child-driven effects of ASB on positive parenting in particular (Anderson et al., 1986; Serbin et al., 2015). The association’s genetic and shared environmental etiology is also consistent with past cross-sectional findings in both middle childhood and adolescence (Di Dio et al., 2025; Ge et al., 1996). Our findings build on this prior cross-sectional work to suggest that the genetic/evocative *r*GE and shared environmental influences continue to underlie associations between youth ASB and maternal positive parenting over time from childhood into adolescence.

Interestingly, these results depart from longitudinal findings in early childhood. Prospective studies from early to middle childhood found the association to be parent-driven and environmental in origin, where children experiencing more positive parenting engaged in less ASB years later, regardless of the child’s genetic liability for ASB (Caspi et al., 2004; Reuben et al., 2016). The inconsistency of these findings with those observed in adolescence suggests that the origins of parenting behavior evolve as children age. Specifically, it has been proposed (see Scarr & McCartney, 1983) that environmental influences decrease and genetic influences increase across adolescence and into adulthood. Indeed, as children age and increasingly engage in autonomy-seeking behaviors, their genetic predispositions may be more fully actualized as they progressively shape their environments with less parental influence. In turn, the impact of the environment on children—including the home environment *and* parents—may become less prominent. This hypothesis has been supported by research on both child ASB (Lyons et al., 1995) and parent-child relationships (McGue et al., 2005; Burt et al., 2024a) and aligns with our findings. Nevertheless, these divergent developmental pathways underscore the need for additional longitudinal, genetically informed, and other experimental studies to be conducted across child development to clarify the mechanisms linking parenting to child ASB.

The importance of this study is enhanced by the strong sampling framework (i.e., birth records), the genetically informed discordant twin design, and the novel integration of both MZ and DZ co-twins into the *same* longitudinal cross-lagged model. Despite its utility for examining the genetic and environmental mechanisms influencing parent-child associations over time, the longitudinal twin differences design has not, to our knowledge, ever been used to contrast MZ and DZ twin differences in the same cross-lagged model. In this study, we demonstrated the value of this innovative approach for facilitating the examination of an association’s direction of effects and etiology within a single, integrated analytical framework.

The present study also has a few limitations. Although our data are MCAR, and we used Maximum Likelihood estimation, which can handle larger amounts of data (Enders, 2001, 2023), we had a moderate amount of missing data in adolescence. Additionally, although the MZ-only and DZ-only models suggest that genetic influences and evocative *r*GE likely underlie the longitudinal association (Scenario 2), the constraint models demonstrated that effects could be constrained to equality across zygosity, findings that suggest the presence of shared environmental confounds (Scenario 3). This finding may reflect the fact that, although statistically significant, the cross-lagged difference-score association in DZ twins was smaller in magnitude. However, this finding could be influenced by the smaller sample of total MZ (*n* = 268) as compared to DZ (*n* = 443) twin pairs. Nonetheless, studies with larger samples would help confirm these possibilities, and additional longitudinal research is needed to replicate this work and further clarify the origins of associations between positive parenting and child ASB.

There are also several limitations of the cross-lagged panel model. Cross-lagged designs may conflate between-person stability with within-person change (e.g., Mund & Nestler, 2019). Our phenotypic cross-lagged models are subject to this limitation, and future studies should examine associations using study designs that can disentangle between- from within-person effects. Notably, however, this limitation does not apply to our twin difference-score analyses. By subtracting one twin’s score from their co-twin’s score, stable between-family influences are removed, so any significant difference-score associations reflect within-family variation that cannot be attributed to family-level confounds. A related limitation is that we utilized only two waves of data that were collected (on average) 7.51 years apart. Cross-lagged models are time-interval dependent, such that the estimated effects are specific to the time interval assessed (e.g., Kuiper & Ryan, 2018). Although phenotypic associations could be constrained across co-twins assessed at Wave 2 *within* versus *after* 7.27 years (i.e., the median lag time) of their initial assessment, it remains possible that these associations *could* vary if the time interval between assessments were shorter or longer than in the present study. Future genetically informed studies should also evaluate positive parenting/ASB associations using additional and more frequent assessments, especially given evidence that evocative *r*GE effects on maternal warmth may not persist to different timescales such as moment-to-moment (i.e., *micro*scale) assessments (Klahr et al., 2013). Nevertheless, our findings shed light on the long-term, dyadic nature of the mother–child relationship at the *macro* timescale in the transition from childhood to adolescence.

We also cannot elucidate the specific genetic influences or shared environmental experience(s) that underlie associations between parenting and ASB. For example, our findings could reflect pleiotropy, where a single gene influences multiple distinct phenotypes (e.g., child ASB and familial relationships). Another possibility is that the genetic factors influencing ASB may be associated with other behaviors that could collectively evoke less positive parenting. In our child-based study, shared environmental influences could reflect familial confounds such as neighborhood characteristics, parental personality traits, or passive *r*GE, whereby children inherit family environments that may be correlated with their genetic predispositions (Klahr & Burt, 2014). Future researchers should illuminate the specific genetic and shared environmental factors driving the longitudinal association between positive parenting and youth ASB.

Our findings are also limited to maternal behaviors and may not extend to paternal behaviors, especially given that child-driven effects on positive parenting, and the etiology of these effects, differ between mothers and fathers (Marceau et al., 2025; Neiderhiser al., 2004; Neiderhiser al., 2007). Relatedly, our measure of positive parenting assessed several behaviors, so we cannot be sure which specific parenting practices are driving our findings. We also utilized a single informant for parenting (twin report) and ASB (maternal report). While this approach avoided shared informant effects, it also potentially limited information since informants provide different yet valuable information due to their context-specific experiences with the child (De Los Reyes & Kazdin, 2005). Future analyses should utilize a multi-informant approach and examine maternal and paternal parenting to extend our understanding of associations between parenting and ASB.

Our archival sample was also selected from moderately-to-severely disadvantaged neighborhoods. Although socioeconomic adversity was not a focus of our study, families in under-resourced neighborhoods typically experience additional stressors that could impact positive parenting, ASB, and the interplay between them (e.g., Burt et al., 2016, 2024a, b). Thus, our findings may not generalize to all socioeconomic contexts. Additionally, while our sample was demographically representative of the state of Michigan at recruitment, an important limitation is that our sample was 82% White. Although positive parenting behaviors (e.g., warmth, support) are consistently associated with less youth ASB across cultural, racial, and ethnic groups (e.g., Rothenberg et al., 2020; Pereyra et al., 2019; Morrison et al., 2019), some studies suggest potential racial/ethnic differences in these associations (e.g., Kang et al., 2023). Future research should thus prioritize examining the etiology of positive parenting/ASB associations in more diverse samples. A final potential limitation is that the children in our study were twins. Although twins are representative of the general population on many traits (Christensen & McGue, 2020), parenting children the same age may present unique challenges for mothers that impact the parenting they provide.

Despite these limitations, our findings have several important implications. First, our results strongly argue against a causal, parent-driven pathway of low levels of positive parenting on youth ASB in the transition from childhood to adolescence. Instead, our results suggest that the association is child-driven, where mothers respond to children’s ASB over time by practicing less positive parenting, and that the association is driven by both evocative *r*GE and, likely to a lesser extent, shared environmental confounds. These findings emphasize the child’s role in shaping the parenting they receive and underscore the importance of considering the child’s influence on parenting behaviors and their outcomes in etiologic models of development.

Our findings also have important implications for the treatment and prevention of youth ASB, as our results for positive parenting differ markedly from those for negative parenting. Prior work indicates that associations between parent–child conflict/harsh parenting and youth ASB are parent-driven and environmental in origin, with children experiencing harsher parenting also tending to engage in more ASB (e.g., Burt et al., 2006). The potential causal effect of harsh parenting supports past experimental treatment studies that consistently demonstrate that reducing harsh/inconsistent discipline (Gardner et al., 2006; Bjørknes et al., 2012; Fossum et al., 2009) or attenuating parent-child conflict (Van Ryzin & Dishion, 2012; Smith et al., 2014) decreases youth ASB. In contrast, the child-driven nature of positive parenting associations may help explain the varied success of current interventions aiming to reduce child ASB by increasing positive parenting. For example, in both childhood and adolescence, improvements in discipline strategies such as praise and positive reinforcement have shown greater success in reducing ASB than have increases in affective behaviors such as communication, involvement, and warmth (e.g., Hagen et al., 2011; Deković et al., 2012; Fagan & Benedini, 2016; Forehand et al., 2014). Interventions may thus benefit from a greater emphasis on the child’s role in influencing the parenting they experience and suggest that interventions may be more effective if they focus on attenuating negative/harsh parenting, rather than increasing positive parenting per se. Indeed, the current results suggest that positive parenting may increase on its own as a function of reductions in youth ASB. Taken together, such inferences highlight the important role behavioral genetic research can play in informing intervention, prevention, and treatment efforts for youth ASB.

## Supplementary Information

Below is the link to the electronic supplementary material.

Supplementary Material 1 (DOCX 207 KB) 

## Data Availability

The data that support the findings of this study are available on request from the corresponding author. The data are not publicly available due to privacy or ethical restrictions by the study IRB.
