# Supplementary material for: Less Positive Parenting Appears to be a Consequence, Rather Than a Cause, of Youth Antisocial Behavior: Results from a Longitudinal Twin Study
Source: Res Child Adolesc Psychopathol. 2026 Feb 14;54(1):32. doi: 10.1007/s10802-026-01425-2 (PMC12906592; doi:10.1007/s10802-026-01425-2)
Supplement: Supplementary file 1 — Supplementary Material 1 (DOCX 207 KB) [file 10802_2026_1425_MOESM1_ESM.docx]

**Supplemental Materials**

**Methods**

**Maternal Positive Parenting**

Observed co-twin differences were relatively large in magnitude. For the full 12-item measure, the mean absolute value of the co-twin difference for positive parenting at Wave 1 was 5.53 (range = 0–27), which corresponds to 94% of the phenotypic variability across the sample (phenotypic *SD* = 5.90, *M* = 23.45, range = 0–32 after setting the scale minimum to zero). Similarly, the mean absolute value of the co-twin difference for this same measure of positive parenting at Wave 2 was 4.85 (range = 0–22), or 79% of the phenotypic variability (phenotypic *SD* = 6.17, *M* = 25.45, range = 0–33 after setting the scale minimum to zero). For the reduced 9-item measure, the mean absolute value of the co-twin difference for positive parenting at Wave 1 was 4.51 (range = 0–21), which corresponds to 92% of the phenotypic variability across the sample (phenotypic *SD* = 4.91, *M* = 19.32, range = 0–26 after setting the scale minimum to zero). Moreover, the mean absolute value of the co-twin difference for this same measure of positive parenting at Wave 2 was 4.13 (range = 0–20), or 81% of the phenotypic variability (phenotypic *SD* = 5.12, *M* = 17.98, range = 0–24 after setting the scale minimum to zero). In other words, average co-twin differences in maternal positive parenting were at least four-fifths the magnitude of average differences among unrelated individuals in the sample.

**Missing Data**

Given the moderate amount of attrition from Wave 1 to Wave 2 (~40%), we evaluated whether our data were missing completely at random (MCAR). We conducted Little’s (1988) MCAR test on the positive parenting and ASB measures at both waves, and the results were non-significant, χ^2^ (18) = 27.20, *p* = .074, indicating that the assumption of data missing at random is tenable. We also examined whether twins who participated only in Wave 1 differed from those who participated in both Waves on positive parenting, child ASB, and socioeconomic status (SES) indices, including parental income and neighborhood disadvantage measures derived from participants’ Wave 1 addresses (i.e., mean Census tract poverty level and an Area Deprivation Index percentile based on national norms). Group differences were tested using independent-samples *t* tests (for continuous measures) and a threshold invariance test (for the categorical parental income variable). Results indicated that twins who participated only at Wave 1 did not significantly differ from those who participated at both Waves on any study or SES measure. See Table S2 for the distribution of parental income and the corresponding invariance test results, and Table S3 for all *t*-test results.

**Table S1**

*Factor Loadings of the PEQ Involvement Scale Items (Child Report) for the Full and Reduced Scales*

|  | Full Scale | | Reduced Scale | |
| --- | --- | --- | --- | --- |
| PEQ Involvement Scale item | Wave 1 | Wave 2 | Wave 1 | Wave 2 |
| I talk about my concerns and my experiences with my mother. | -0.35 | -0.65 | -0.33 | -0.65 |
| My mother praises me when I do something well. | -0.30 | -0.50 | – | – |
| My mother doesn’t know my hobbies. | 0.35 | 0.54 | – | – |
| I don’t want my friends to meet my mother. | 0.35 | 0.55 | 0.35 | 0.55 |
| My mother often comforts me when I am discouraged or have had a disappointment. | -0.36 | -0.53 | -0.32 | -0.51 |
| My mother and I don’t have much to talk about when we are together. | 0.50 | 0.68 | 0.53 | 0.68 |
| My mother tries to keep up with how well I do in school and/or in my job. | -0.33 | -0.45 | – | – |
| I prefer not to talk about my personal problems with my mother. | 0.46 | 0.63 | 0.48 | 0.65 |
| My mother doesn’t seem to know much about how I do in school. | 0.47 | 0.58 | 0.46 | 0.56 |
| My mother and I do not do a lot of things together. | 0.44 | 0.70 | 0.45 | 0.70 |
| I don’t feel very close to my mother. | 0.52 | 0.77 | 0.51 | 0.78 |
| My mother doesn’t know much about how I spend my time. | 0.47 | 0.70 | 0.48 | 0.70 |

*Note.* Standardized factor loadings for each item of the Parent-Child Involvement Scale on the Parental Environment Questionnaire (PEQ) are presented for the full 12-item scale and a reduced 9-item scale derived from confirmatory factor analyses (CFA) that best fit the observed data at Waves 1 (in middle childhood) and 2 (in adolescence). Loadings represent those of the raw, non-recoded scale items answered on a scale of 1 = *definitely true* to 4 = *definitely false*. Dashes indicate items that were excluded from the reduced scale due to comparatively lower factor loadings. Fit statistics for all CFAs: full scale Wave 1: χ^2^ (54) = 177.40, *p* < .001, root mean square error of approximation (RMSEA) = 0.04, comparative fit index (CFI) = 0.90, standardized root mean square residual (SRMR) = 0.04; full scale Wave 2: χ^2^ (54) = 222.55, *p* < .001, RMSEA = 0.06, CFI = 0.92, SRMR = 0.04; reduced scale Wave 1: χ^2^ (27) = 67.82, *p* < .001, RMSEA = 0.03, CFI = 0.95, SRMR = 0.03; reduced scale Wave 2: χ^2^ (27) = 104.68, *p* < .001 RMSEA = 0.06, CFI = 0.95, SRMR = 0.04. Factor loadings in all models were significant at *p* < .001.

**Table S2**

*Distribution of Parental Income Presented Separately for Twins Who Participated in Both Waves and Twins Who Participated Only in Wave 1*

| Variable | Participation in Wave 1 only | Participation in Wave 1  and Wave 2 |
| --- | --- | --- |
| Parental income |  |  |
| <$10,000 | 20 (3.70%) | 24 (3.00%) |
| $10,000–$20,000 | 54 (10.10%) | 60 (7.40%) |
| $20,000–$30,000 | 66 (12.40%) | 74 (9.10%) |
| $30,000–$40,000 | 48 (9.00%) | 80 (9.90%) |
| $40,000–$50,000 | 86 (16.10%) | 114 (14.10%) |
| >$50,000 | 260 (48.70%) | 458 (56.50%) |
| Model comparison |  |  |
| Threshold invariance test | Δχ^2^ (5) = 10.33, *p* = .067 | |

*Note.* Percentages reflect proportions within each sample. The chi-square difference test (Δχ² (*df*)) compared a saturated model (with freed thresholds) to a restricted model with thresholds constrained to be equal across the two groups to evaluate whether the distribution of parental income significantly differed between those twins who participated in both Waves versus those participating in Wave 1 only.

**Table S3**

*Results of Independent-Samples t Tests Evaluating Differences Between Twins Who Participated in Both Waves and Twins Who Participated Only in Wave 1 on Study and SES Measures*

|  | Mean (*SD*) | | |  | |  |  |  |
| --- | --- | --- | --- | --- | --- | --- | --- | --- |
| Variable | | Wave 1 only | Waves 1 and 2 | | *t* | df | *p* | Cohen’s D |
| Study measures | |  |  |  | |  |  |  |
| Wave 1 PP (12-item) | | 39.69 (5.84) | 39.29 (5.94) | 1.24 | | 1378 | .214 | 0.07 |
| Wave 1 PP (9-item) | | 29.45 (4.80) | 29.23 (4.98) | 0.82 | | 1378 | .413 | 0.05 |
| Wave 1 ASB | | 6.51 (7.22) | 6.12 (6.36) | 1.05 | | 1106 | .295 | 0.06 |
| SES characteristics | |  |  |  | |  |  |  |
| Mean Census tract poverty level at Wave 1 | | 0.20 (0.15) | 0.20 (0.14) | 0.96 | | 1410 | .335 | 0.05 |
| ADI | | 64.14 (21.00) | 62.47 (20.41) | 1.49 | | 1394 | .137 | 0.08 |

*Note.* Results of *t* tests comparing maternal Wave 1 positive parenting (PP; 12- and 9-item versions), child antisocial behavior (ASB), Census-tract defined poverty levels, and Area Deprivation Index (ADI) scores (as measured by percentiles at the national level) between twins who participated in Wave 1 only (in middle childhood) to those who participated in Waves 1 and 2 (in middle childhood and adolescence). Means and standard deviations (*SD*) for the raw positive parenting and raw, non-log-transformed ASB data before regressing out participants’ sex assigned at birth were tested. SES = socioeconomic status.

**Table S4**

*Phenotypic and Twin Difference Correlations Between Maternal Positive Parenting (12-Item Scale) and Youth ASB in the Full, Unselected Sample*

| Measure | Wave 1 ASB | Wave 2 ASB | Wave 1 PP | Wave 2 PP |
| --- | --- | --- | --- | --- |
| Individual level – Phenotypic associations | | | | |
| 1. Wave 1 ASB | 1 |  |  |  |
| 2. Wave 2 ASB | 0.41** | 1 |  |  |
| 3. Wave 1 PP | -0.09** | -0.05 | 1 |  |
| 4. Wave 2 PP | -0.11** | -0.26** | 0.18** | 1 |
| Family level – Twin difference-score associations^a^ | | | | |
| MZ Twins | | | | |
| 1. Wave 1 ASB | 1 |  |  |  |
| 2. Wave 2 ASB | 0.21** | 1 |  |  |
| 3. Wave 1 PP | -0.09 | 0.01 | 1 |  |
| 4. Wave 2 PP | -0.01 | -0.23** | 0.05 | 1 |
| DZ Twins | | | | |
| 1. Wave 1 ASB | 1 |  |  |  |
| 2. Wave 2 ASB | 0.37** | 1 |  |  |
| 3. Wave 1 PP | -0.07 | -0.06 | 1 |  |
| 4. Wave 2 PP | -0.15* | -0.18** | 0.14* | 1 |

*Note.* Wave 1 maternal positive parenting (PP; 12-item version) and antisocial behavior (ASB) data were collected in middle childhood, while Wave 2 data were collected in adolescence. All phenotypic and twin difference-score correlations were conducted between positive parenting and log-transformed ASB variables with sex regressed out. Correlation is significantly greater than zero: ** *p* < .01 and * *p* < .05, two-tailed.

^a^Correlation analyses were conducted on signed difference scores.

**Table S5**

*Descriptive Statistics for Maternal Positive Parenting and ASB Measures Across the Full Sample and Separately Within MZ and DZ Twins*

|  | Individual scores | | | | | Absolute twin difference scores^a^ | | | | | |  |
| --- | --- | --- | --- | --- | --- | --- | --- | --- | --- | --- | --- | --- |
| Measure | *M* | *SD* | Min | Max | *n* | | *M* | *SD* | Min | Max | *n* |  |
| Full Sample | | | | | | | | | | | |  |
| 1. Wave 1 ASB | 6.28* | 6.72 | 0 | 41 | 1409 | | 4.46* | 5.30 | 0 | 30 | 704 |  |
| 2. Wave 2 ASB | 3.57 | 5.02 | 0 | 47 | 840 | | 3.19 | 4.85 | 0 | 39 | 420 |  |
| 3. Wave 1 PP (12-item) | | 39.45* | 5.90 | 16 | 48 | 1380 | | 5.53* | 4.53 | 0 | 27 | 684 |
| 4. Wave 2 PP (12-item) | 40.45 | 6.17 | 15 | 48 | 779 | | 4.85 | 4.12 | 0 | 22 | 384 |  |
| 3. Wave 1 PP (9-item) | 29.32* | 4.91 | 10 | 36 | 1380 | | 4.51 | 3.76 | 0 | 21 | 684 |  |
| 4. Wave 2 PP (9-item) | 29.98 | 5.12 | 12 | 36 | 779 | | 4.13 | 3.45 | 0 | 20 | 384 |  |
| MZ Twins | | | | | | | | | | | |  |
| 1. Wave 1 ASB | 5.47* | 5.82 | 0 | 37 | 535 | | 3.12† | 3.89 | 0 | 25 | 267 |  |
| 2. Wave 2 ASB | 2.89 | 4.83 | 0 | 47 | 324 | | 2.48† | 4.85 | 0 | 39 | 162 |  |
| 3. Wave 1 PP (12-item) | 39.20 | 5.69 | 20 | 48 | 525 | | 5.27* | 4.00 | 0 | 21 | 261 |  |
| 4. Wave 2 PP (12-item) | 39.77 | 6.82 | 15 | 48 | 288 | | 4.24† | 3.51 | 0 | 17 | 142 |  |
| 3. Wave 1 PP (9-item) | 29.13 | 4.68 | 13 | 36 | 525 | | 4.22* | 3.40 | 0 | 18 | 261 |  |
| 4. Wave 2 PP (9-item) | 29.41 | 5.67 | 12 | 36 | 288 | | 3.52† | 3.02 | 0 | 13 | 142 |  |
| DZ Twins | | | | | | | | | | | |  |
| 1. Wave 1 ASB | 6.77* | 7.17 | 0 | 41 | 874 | | 5.27*† | 5.85 | 0 | 30 | 437 |  |
| 2. Wave 2 ASB | 4.00 | 5.10 | 0 | 39 | 516 | | 3.63† | 4.80 | 0 | 33 | 258 |  |
| 3. Wave 1 PP (12-item) | 39.60* | 6.03 | 16 | 48 | 855 | | 5.69 | 4.82 | 0 | 27 | 423 |  |
| 4. Wave 2 PP (12-item) | 40.85 | 5.73 | 20 | 48 | 491 | | 5.21† | 4.41 | 0 | 22 | 242 |  |
| 3. Wave 1 PP (9-item) | 29.43* | 5.04 | 10 | 36 | 855 | | 4.69 | 3.97 | 0 | 21 | 423 |  |
| 4. Wave 2 PP (9-item) | 30.31 | 4.74 | 12 | 36 | 491 | | 4.48† | 3.64 | 0 | 20 | 242 |  |

*Note.* Min = minimum; max = maximum; MZ = Monozygotic; DZ = Dizygotic; Wave 1 maternal positive parenting (PP) and antisocial behavior (ASB) data were collected in middle childhood, while Wave 2 data were collected in adolescence. Descriptives for raw positive parenting (for both the full 12-item and the reduced 9-item versions) and raw, non-log-transformed ASB data are presented before regressing out participants’ sex assigned at birth.

^a^Twin difference scores are presented in absolute value form to emphasize the true magnitude of twin differences. However, in order to examine the direction of any significant effects, we conducted our final analyses on signed difference scores.

*Indicates a within-trait change in phenotypic or sibling difference scores across age at *p* < .05.

†Indicates a significant difference between MZ and DZ co-twin differences in the trait at *p* < .05.

**Table S6**

*Phenotypic and Twin Difference Correlations Between Maternal Positive Parenting and Youth ASB in the Full, Unselected Sample*

| Measure | Wave 1 ASB | Wave 2 ASB | Wave 1 PP | Wave 2 PP |
| --- | --- | --- | --- | --- |
| Individual level – Phenotypic associations | | | | |
| 1. Wave 1 ASB | 1 |  |  |  |
| 2. Wave 2 ASB | 0.41** | 1 |  |  |
| 3. Wave 1 PP | -0.10** | -0.06 | 1 |  |
| 4. Wave 2 PP | -0.11** | -0.26** | 0.14** | 1 |
| Family level – Twin difference-score associations^a^ | | | | |
| MZ Twins | | | | |
| 1. Wave 1 ASB | 1 |  |  |  |
| 2. Wave 2 ASB | 0.21** | 1 |  |  |
| 3. Wave 1 PP | -0.05 | -0.02 | 1 |  |
| 4. Wave 2 PP | -0.01 | -0.23** | 0.07 | 1 |
| DZ Twins | | | | |
| 1. Wave 1 ASB | 1 |  |  |  |
| 2. Wave 2 ASB | 0.37** | 1 |  |  |
| 3. Wave 1 PP | -0.09 | -0.07 | 1 |  |
| 4. Wave 2 PP | -0.15* | -0.20** | 0.08 | 1 |

*Note.* Wave 1 maternal positive parenting (PP; 9-item version) and antisocial behavior (ASB) data were collected in middle childhood, while Wave 2 data were collected in adolescence. All phenotypic and twin difference-score correlations were conducted between positive parenting and log-transformed ASB variables with sex regressed out. Correlation is significantly greater than zero: ** *p* < .01 and * *p* < .05, two-tailed.

^a^Correlation analyses were conducted on signed difference scores.

**Figure S1**

*Cross-lagged Model of the Association Between Maternal Positive Parenting and Youth ASB From Middle Childhood to Adolescence*

e1

*b*2 (SE)

*r*2 (SE)

*b*3 (SE)

*b*1 (SE)

Wave 1

Positive Parenting

Wave 1 ASB

Wave 2

Positive Parenting

Wave 2 ASB

*r*1 (SE)

*b*4 (SE)

e2

*Note.* Cross-lagged model of the association between antisocial behavior (ASB) and maternal positive parenting from middle childhood (Wave 1) to adolescence (Wave 2). Cross-age paths are partial regression coefficients and are indicated with a “*b*” followed by one numeral. Within-age correlations are indicated by a “*r*” followed by a numeral. The residual variance in positive parenting and ASB in adolescence is represented by an “*e*” followed by a single numeral.

**Figure S2**

*Hypothetical Twin Difference Correlation Results for the Association Between Maternal Positive Parenting and ASB*

*Note.* In Scenario 1, the association is purely environmental in origin. In Scenario 2, the association is attributable to genetic influences. In Scenario 3, the association is both genetic and shared environmental in origin. DZ = Dizygotic; MZ = Monozygotic.

**Figure S3**

*Phenotypic Cross-Lagged Model of Maternal Positive Parenting (12-Item Scale) and Youth ASB*

**-0.09 (0.04)***

**-0.21 (0.04)****

**0.16 (0.04)****

-0.01 (0.03)

Wave 1

Positive Parenting

Wave 1 ASB

Wave 2

Positive Parenting

Wave 2 ASB

**-0.09 (0.03)****

**0.40 (0.04)****

*Note. N* = 1,422. Fit indices: χ^2^ (0) = 0.00, *p* = .000, root mean square error of approximation (RMSEA) = 0.00, comparative fit index (CFI) = 1.00, standardized root mean square residual (SRMR) = 0.00. The observed variables are maternal positive parenting (12-item version) and log-transformed youth ASB scores across the full sample at Wave 1 (middle childhood) and Wave 2 (adolescence). Unstandardized regression coefficients are presented for the single-headed arrows (i.e., paths), and correlations are presented for the double-headed arrows. Standard errors for the estimates are presented in parentheses. Significant estimates are bolded. ** *p* < .01 and * *p* < .05, two-tailed.

**Figure S4**

*Cross-Lagged Model of Twin Differences in Maternal Positive Parenting (12-Item Scale) and Twin Differences in Youth ASB in MZ and DZ Twin Pairs*

-0.001 (0.09)/

**-0.15 (0.06)***

**-0.17 (0.06)**/**

**-0.15 (0.07)***

0.04 (0.06)/

0.11 (0.06)

0.02 (0.06)/

-0.03 (0.05)

Wave 1

Positive Parenting

Wave 1 ASB

Wave 2

Positive Parenting

Wave 2 ASB

-0.08 (0.06)/

-0.10 (0.07)

**0.23 (0.08)**/**

**0.37 (0.06)****

*Note.* Monozygotic (MZ) *N* = 268 pairs; Dizygotic (DZ) *N* = 443 pairs. Fit indices for both the MZ and DZ twin difference-score models: χ^2^ (0) = 0.00, *p* = .000, root mean square error of approximation (RMSEA) = 0.00, comparative fit index (CFI) = 1.00, standardized root mean square residual (SRMR) = 0.00. The observed variables are MZ and DZ twin difference scores in maternal positive parenting (12-item version) and log-transformed youth ASB at Wave 1 (middle childhood) and Wave 2 (adolescence). Path and correlation estimates are presented first for the MZ model and then just below for the DZ model (MZ estimate (*SE*) / DZ estimate (*SE*)). Unstandardized regression coefficients are presented for the single-headed arrows (i.e., paths), and correlations are presented for the double-headed arrows. Standard errors for the estimates are presented in parentheses. Significant estimates are bolded. ** *p* < .01 and * *p* < .05, two-tailed.

**Figure S5**

*Cross-lagged Constraint Model of Twin Differences in Maternal Positive Parenting (12-Item Scale) and Twin Differences in Youth ASB*

***-0.10 (0.05)***†^a^

**-0.17 (0.05)****

0.07 (0.04)

-0.003 (0.04)

Wave 1

Positive Parenting

Wave 1 ASB

Wave 2

Positive Parenting

Wave 2 ASB

**-0.09 (0.04)***

**0.32 (0.05)****

*Note. N* = 711 pairs; Monozygotic (MZ) *n* = 268 pairs, Dizygotic (DZ) *n* = 443 pairs. Fit indices: χ^2^ (6) = 4.83, *p* = .566, root mean square error of approximation (RMSEA) = 0.00, comparative fit index (CFI) = 1.00, standardized root mean square residual (SRMR) = 0.03. The observed variables are MZ and DZ twin difference scores in maternal positive parenting (12-item version) and log-transformed youth ASB at Wave 1 (middle childhood) and Wave 2 (adolescence). Unstandardized regression coefficients are presented for the single-headed arrows (i.e., paths), and correlations are presented for the double-headed arrows. Standard errors for the estimates are presented in parentheses. Significant estimates are bolded. ** *p* < .01, * *p* < .05, and † *p* < .10, two-tailed.

^a^*p* = .060.

**Figure S6**

*Cross-Lagged Model of Twin Differences in the Maternal Positive Parenting (12-Item Scale) and Twin Differences in Youth ASB for MZ and DZ Twin Pairs Most Discordant on ASB (top 25%)*

-0.04 (0.13)/

**-0.25 (0.08)****

-0.16 (0.17)/

**-0.30 (0.15)***

0.18 (0.15)/

0.14 (0.10)

-0.13 (0.16)/

-0.04 (0.10)

Wave 1

Positive Parenting

Wave 1 ASB

Wave 2

Positive Parenting

Wave 2 ASB

**-0.53 (0.27)***/

-0.18 (0.16)

**0.28 (0.13)*/**

**0.37 (0.08)****

*Note.* Monozygotic (MZ) *N* = 49 pairs; Dizygotic (DZ) *N* = 147 pairs. Fit indices for both the MZ and DZ twin difference-score models: χ^2^ (0) = 0.00, *p* = .000, root mean square error of approximation (RMSEA) = 0.00, comparative fit index (CFI) = 1.00, standardized root mean square residual (SRMR) = 0.00. The observed variables are MZ and DZ twin difference scores in maternal positive parenting (12-item version) and log-transformed youth antisocial behavior (ASB) at Wave 1 (middle childhood) and Wave 2 (adolescence). Path and correlation estimates are presented first for the MZ model and then just below for the DZ model (MZ estimate (*SE*) / DZ estimate (*SE*)). Unstandardized regression coefficients are presented for the single-headed arrows (i.e., paths), and correlations are presented for the double-headed arrows. Significant estimates are bolded. ** *p* < .01 and * *p* < .05, two-tailed.

**Figure S7**

*Cross-Lagged Constraint Model of Twin Differences in Maternal Positive Parenting (12-Item Scale) and Twin Differences in Youth ASB for Twin Pairs Most Discordant on ASB (top 25%)*

**-0.20 (0.07)****

**-0.25 (0.12)***

0.13 (0.08)

-0.06 (0.09)

Wave 1

Positive Parenting

Wave 1 ASB

Wave 2

Positive Parenting

Wave 2 ASB

**-0.29 (0.14)***

**0.36 (0.07)****

*Note.* *N* = 196 pairs; Monozygotic (MZ) *n* = 49 pairs, Dizygotic (DZ) *n* = 147 pairs. Fit indices: χ^2^ (6) = 4.04, *p* = .672, root mean square error of approximation (RMSEA) = 0.00, comparative fit index (CFI) = 1.00, standardized root mean square residual (SRMR) = 0.05. The observed variables are MZ and DZ twin difference scores maternal positive parenting (12-item version) and log-transformed youth ASB at Wave 1 (middle childhood) and Wave 2 (adolescence). Unstandardized regression coefficients are presented for the single-headed arrows (i.e., paths), and correlations are presented for the double-headed arrows. Standard errors for the estimates are presented in parentheses. Significant estimates are bolded. ** *p* < .01 and * *p* < .05, two-tailed.

**Figure S8**

*Cross-lagged Constraint Model of Maternal Positive Parenting (12-Item Scale) and Youth ASB*

*for Co-Twins Assessed at Wave 2 Within or After 7.27 Years of Their Initial Assessment*

**-0.09 (0.04)***

**-0.21 (0.03)****

**0.17 (0.04)****

-0.01 (0.03)

Wave 1

Positive Parenting

Wave 1 ASB

Wave 2

Positive Parenting

Wave 2 ASB

-0.04 (0.04)

**0.41 (0.03)****

*Note. N* = 852; Co-twins assessed at Wave 2 (in adolescence) within 7.27 years of their Wave 1 assessment (in middle childhood) *n* = 418, co-twins assessed at Wave 2 after 7.27 years of their Wave 1 assessment *n* = 434. Fit indices: χ^2^ (6) = 7.81, *p* = .252, root mean square error of approximation (RMSEA) = 0.03, comparative fit index (CFI) = 0.99, standardized root mean square residual (SRMR) = 0.03. The observed variables are maternal positive parenting (12-item version) and log-transformed youth ASB scores across the full sample at Wave 1 and Wave 2. Unstandardized regression coefficients are presented for the single-headed arrows (i.e., paths), and correlations are presented for the double-headed arrows. Standard errors for the estimates are presented in parentheses. Significant estimates are bolded. ** *p* < .01 and * *p* < .05, two-tailed.

**Figure S9**

*Cross-Lagged Model of Twin Differences in the Maternal Positive Parenting and Twin Differences in Youth ASB for MZ and DZ Twin Pairs Most Discordant on ASB (top 25%)*

-0.04 (0.12)/

**-0.26 (0.08)****

-0.24 (0.17)/

**-0.36 (0.16)***

0.18 (0.16)/

0.13 (0.11)

-0.18 (0.17)/

-0.04 (0.10)

Wave 1

Positive Parenting

Wave 1 ASB

Wave 2

Positive Parenting

Wave 2 ASB

-0.35(0.23)/

-0.26 (0.16)

**0.28 (0.12)*/**

**0.37 (0.08)****

*Note.* Monozygotic (MZ) *N* = 49 pairs; Dizygotic (DZ) *N* = 147 pairs. Fit indices for both the MZ and DZ twin difference-score models: χ^2^ (0) = 0.00, *p* = .000, root mean square error of approximation (RMSEA) = 0.00, comparative fit index (CFI) = 1.00, standardized root mean square residual (SRMR) = 0.00. The observed variables are MZ and DZ twin difference scores in maternal positive parenting (9-item version) and log-transformed youth antisocial behavior (ASB) at Wave 1 (middle childhood) and Wave 2 (adolescence). Path and correlation estimates are presented first for the MZ model and then just below for the DZ model (MZ estimate (*SE*) / DZ estimate (*SE*)). Unstandardized regression coefficients are presented for the single-headed arrows (i.e., paths), and correlations are presented for the double-headed arrows. Significant estimates are bolded. ** *p* < .01 and * *p* < .05, two-tailed.

**Figure S10**

*Cross-lagged Constraint Model of Maternal Positive Parenting (9-item) and Youth ASB*

*for Co-Twins Assessed at Wave 2 Within or After 7.27 Years of Their Initial Assessment*

**-0.09 (0.04)****

**-0.20 (0.03)****

**0.14 (0.04)****

-0.02 (0.03)

Wave 1

Positive Parenting

Wave 1 ASB

Wave 2

Positive Parenting

Wave 2 ASB

-0.04 (0.03)

**0.41 (0.03)****

*Note. N* = 852; Co-twins assessed at Wave 2 (in adolescence) within 7.27 years of their Wave 1 assessment (in middle childhood) *n* = 418, co-twins assessed at Wave 2 after 7.27 years of their Wave 1 assessment *n* = 434. Fit indices: χ^2^ (6) = 7.10, *p* = .312, root mean square error of approximation (RMSEA) = 0.02, comparative fit index (CFI) = 1.00, standardized root mean square residual (SRMR) = 0.02. The observed variables are maternal positive parenting (9-item version) and log-transformed youth ASB scores across the full sample at Wave 1 and Wave 2. Unstandardized regression coefficients are presented for the single-headed arrows (i.e., paths), and correlations are presented for the double-headed arrows. Standard errors for the estimates are presented in parentheses. Significant estimates are bolded. ** *p* < .01 and * *p* < .05, two-tailed.

**References**

Little, R. J. A. (1988). A test of missing completely at random for multivariate data with missing values. *Journal of the American Statistical Association*, *83*(404), 1198–1202. https://doi.org/10.1080/01621459.1988.10478722
